# Supplementary figures and images for: Establishing an ecological security pattern for urban agglomeration, taking ecosystem services and human interference factors into consideration
Source: PeerJ. 2019 Jul 15;7:e7306. doi: 10.7717/peerj.7306 (PMC6637928; doi:10.7717/peerj.7306)

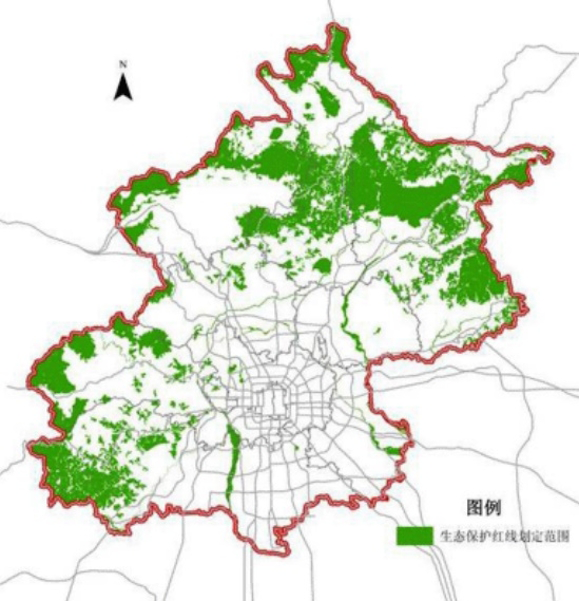

Supplement: Supplemental Information 27 — The basic ecological red line pattern in Beijing is “two screens and two belts,” the green part of the data is the location of the ecological red line. [file peerj-07-7306-s027.png]

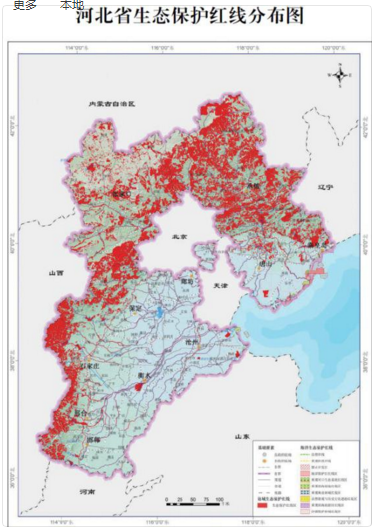

Supplement: Supplemental Information 28 — The basic ecological red line pattern in Hebei Province is “two screens, two belts and multiple points,” the red part of the data is the location of the ecological red line. [file peerj-07-7306-s028.png]

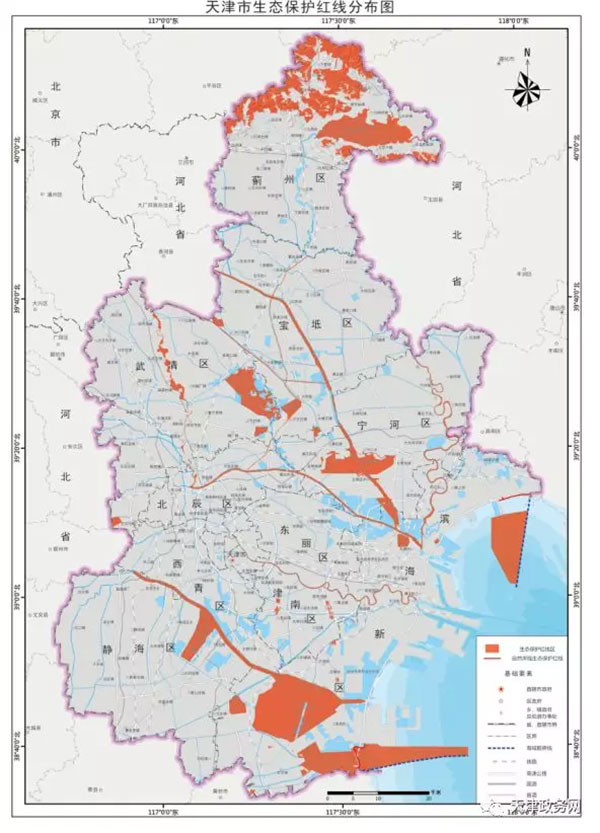

Supplement: Supplemental Information 29 — The basic pattern of the ecological protection red line space in Tianjin is “three zones, one zone and more points,” the red part of the data is the location of the ecological red line. [file peerj-07-7306-s029.png]
